# Prognostic factors of cataract surgery in patients with uveitis

**DOI:** 10.1007/s10792-023-02860-6

**Published:** 2023-09-12

**Authors:** Kristina Jevnikar, Saša Počkar, Lan Umek, Aniki Rothova, Natasa Vidovic Valentincic

**Affiliations:** 1 Department of Ophthalmology, University Medical Centre Ljubljana, Grablovičeva 46, 1000 Ljubljana, Slovenia; 2 Faculty of Medicine, University of Ljubljana, Ljubljana, Slovenia; 3 Manchester Royal Eye Hospital, Manchester University Hospitals NHS Foundation Trust, Manchester Academic Health Science Centre, Manchester, UK; 4 Faculty of Public Administration, University of Ljubljana, Ljubljana, Slovenia; 5 Department of Ophthalmology, Erasmus MC, University Medical Center Rotterdam, Rotterdam, The Netherlands

**Keywords:** Cataract, Uveitis, Phacoemulsification, Cystoid macular edema, Complications, Outcome

## Abstract

**Purpose:**

To evaluate the long-term visual outcomes of patients with uveitis undergoing cataract surgery and to identify possible factors influencing the visual prognosis and the development of postoperative complications.

**Methods:**

Retrospective study of all patients with uveitis who underwent cataract surgery between January 2015 and February 2020 in our tertiary referral center.

**Results:**

A total of 78 eyes from 78 patients were included in the study. The best-corrected visual acuity (BCVA) improved in 86% of patients, and a BCVA of 0.5 or better was achieved in 57 (73%) patients. A significant correlation was shown between the preoperative and postoperative BCVA (Spearman *r* = 0.521, *p* < 0.01). Final BCVA differed between diverse anatomical uveitis entities (*p* = 0.047), and anterior uveitis demonstrated the best outcomes. Chronic uveitis resulted in a worse final BCVA than acute recurrent uveitis (*p* = 0.001). The presence of CME any time before the surgery and intermediate uveitis were associated with worse visual prognosis, while systemic therapy for uveitis before surgery and iris manipulation during surgery were not related to visual outcomes. Postoperative development of cystoid macular edema (CME) was closely associated with preexisting CME (*p* < 0.001) and intermediate uveitis (*p* = 0.01).

**Conclusions:**

Visual results of cataract surgery in patients with uveitis were beneficial, but limited visual outcomes were more frequently observed in patients with chronic uveitis and intermediate uveitis with a history of CME. In consequence, prevention, or adequate treatment of CME, especially in patients with intermediate uveitis, might result in better visual results of their cataract surgery.

## Introduction

A cataract is an important cause of visual impairment in patients with uveitis. It develops due to a combination of prolonged inflammation and treatment with corticosteroids and affects 50–70% of patients [1–3]. Compared to age-related cataracts, patients with uveitis tend to be younger and often exhibit co-morbidity such as cystoid macular edema (CME) and elevated intraocular pressure (IOP) [4–8]. Therefore, the final best-corrected visual acuity (BCVA) tends to be worse, and the prognosis is generally more guarded [5]. Furthermore, the surgery is frequently challenging due to miotic pupil, posterior synechiae, iris atrophy, zonular weakness, and pupillary membrane [1, 4, 9, 10]. The most common postoperative complications include posterior capsule opacification, CME, recurrent or persistent inflammation, glaucoma, cyclitic and/ or epiretinal membrane development, and IOL dislocation [1, 4, 9–13]. It has been shown that delaying cataract surgery until best-corrected visual acuity (BCVA) significantly deteriorates may not be advisable. [5] Since the younger population is affected, a good postoperative outcome is crucial to diminish the disease burden. It is known that careful management of patients and well-controlled inflammation, which needs to be quiet for at least three months before the surgery, are essential for a desirable outcome [2, 3, 8, 9, 13, 14]. Nevertheless, postoperative outcomes may vary, and the underlying reasons are still not completely understood. This study aimed to evaluate the visual outcomes of patients with various etiologies of uveitis undergoing cataract surgery and to identify possible factors influencing the visual outcomes and the development of postoperative complications.

## Methods

### Data collection

All 78 consecutive adult patients with uveitis who underwent cataract surgery (41 females, 37 males) by a single surgeon at Eye Hospital, University medical center Ljubljana, Slovenia (serving as a tertiary referral center) between January 2015 and February 2020 were included in this retrospective observational study. Inclusion criteria were a diagnosis of uveitis and a cataract surgery performed at our tertiary referral center. The following exclusion criteria were applied: concomitant ocular diseases, systemic treatment linked to retinal toxicity, high myopia (> − 6) and high hyperopia (> + 5). The Slovenian medical ethics committee approved the study (0120-346/2021/3). Patient’s medical records were reviewed, and the following data were retrieved: age, gender, pre-existing co-morbidity, etiology, and anatomical localization of uveitis according to Standardization of Uveitis Nomenclature diagnostic criteria, systemic medications, BCVA according to Snellen chart, intraocular pressure (IOP), type of surgery and postoperative complications. The follow-up data were collected at 1 month, 6 months, 1 year, and at three years after surgery. Complications related to surgery were considered to occur within 6 months after the intervention. We registered all complications of cataract surgery, specifically intraoperative posterior capsule rupture, postoperative presence of CME, glaucoma, retinal detachment, and other major postoperative changes. CME was defined as the presence of cystoid cavities always evaluated with swept-source optical coherence tomography (SS-OCT, Topcon DRI OCT Triton; Topcon Corp., Tokyo, Japan. Transient IOP elevation, which normalized within 2 weeks, was registered but not classified as a major complication. The presence of PCO was not registered.

## Peri- and postoperative protocol

All patients received a topical dexamethasone 0.1% (Dexamono®) and a nonsteroidal anti-inflammatory drug (NSAID)—nepafenac 0.1% (Nevanac®) 3 times daily three days before the surgery according to the guidelines. Patients on systemic uveitis treatment remained on their regular dosages if the disease was quiet for more than 6 months before the surgery. Intravenous corticosteroids (500 mg of methylprednisolone daily, starting 1 day before surgery, 3 days in total) were added to the treatment in 5 patients who exhibited active inflammation in six months prior to surgery (which could not be postponed due to poor fundus visibility). Out of these 5 patients 2 exhibited sarcoidosis, 2 idiopathic intermediary uveitis and 1 Behcet’s disease. Standard cataract surgery was defined as phacoemulsification with lens implantation in topical anesthesia. A 2.2 mm main temporal clear corneal incision and one or more side-port corneal incisions were made. Small pupils and posterior synechiae were managed with synechiolysis, iris retractors, or mechanical devices (Malyugin ring®) as needed. Trypan blue (VisionBlue®) was used to stain the anterior capsule in white cataracts. A 5 mm anterior continuous curvilinear capsulorhexis was followed by hydro-dissection, phacoemulsification, and IOL implantation. A hydrophobic intraocular lens was implanted. Dexamethasone implant (700 μg, Ozurdex) was administered during the surgery in 9 patients with chronic or recurrent CME prior to surgery. Preoperative posterior capsule rupture was seen in 2 (3%) patients. Routine postoperative treatment consisted of 0.1% dexamethasone phosphate 10 times daily and tapered slowly over three months and nepafenac 0.1% three times used for three months. Final BCVA was defined as BCVA at three years follow-up.

## Statistical analysis

Only one eye per patient was included in the analysis (first cataract surgery). Descriptive statistics included mean ± standard deviation (SD) or median with the range as appropriate. Continuous parameters were analyzed using the Mann–Whitney *U* test and Kruskal–Wallis *H* test, whereas categorical parameters were analyzed using the chi-squared test. The Wilcoxon sign rank test was used for a change in BCVA after surgery. For the prediction of the final BCVA based on other assessed parameters, linear regression was used. All calculations on BCVA were performed after conversion to the logMAR scale. Values corresponding to counting fingers, hand movement, light perception, and no light perception were substituted with 2.10, 2.40, 2.70, and 3.00 logMAR units. Kaplan-Maier curve was used to estimate the time to the postoperative occurrence of CME. *p* ≤ 0.05 was considered statistically significant. Python 3.8.5 was used for the statistical analyses.

## Results

### Baseline demographics and clinical characteristics

Seventy-eight patients who underwent cataract surgery by a single surgeon at the department of Ophthalmology, University medical center Ljubljana, a tertiary referral center between January 2015 and February 2020, were included. Baseline demographics and clinical characteristics are presented in Table 1. The most common anatomical location of uveitis was intermediate uveitis. At the time of surgery, 23 patients (30%) were receiving systemic immunosuppressive medication. Preoperative CME was diagnosed in 20 (26%) patients, and in 9 (12%) in the six months before the surgery (those patients received perioperative intraocular dexamethasone). Preexisting macular abnormalities (in addition to CME) were present in 10 (13%) patients (6 with epimacular membrane, 4 with age-related macular degeneration). Standard cataract surgery was performed in 17 (22%) patients, and 50 (64%) patients required an additional iris procedure. The patients who received dexamethasone implant exhibited as expected, a higher rate of preoperative CME in comparison with the remaining patients (*p* < 0.0001).

**Table 1:** Baseline characteristics of patients with uveitis

	No. of patients*	%
*Total number of patients*	78	100
Gender		
Male	37	47
Female	41	53
Age		
Average (SD)	58.8 (17.2)	
Median (range)	59.5 (20–84)	
Course of uveitis		
Chronic	55	71
Recurrent	23	29
Anatomical localization		
Anterior	27	35
Intermediate	37	47
Posterior	6	8
Panuveitis	8	10
Etiology		
Idiopathic	45	58
HLA B27-associated	8	10
Herpetic anterior uveitis	8	10
Fuchs Heterochromic Uveitis	5	6
Sarcoidosis-associated	5	6
Miscellaneous**	7	9
	13	17
*Preexsisting CME*		
Systemic immunosuppression		
None	55	71
DMARD and antimetabolic drugs	17	22
DMARD + Biologics	6	8
*Type of surgery*		
Phaco + IOL	17	22
Phaco + IOL + iris procedure***	50	64
Phaco + IOL + intravitreal dexamethasone	9	12

*DMARD* disease-modifying antirheumatic drugs, *Phaco* phacoemulsification, *IOL* intraocular lens

^*^One eye per patient included, specifically the first eye undergoing cataract surgery

^**^Includes Behcet’s disease (*n* = 2), juvenile idiopathic arthritis-associated uveitis (*n* = 3), toxoplasmosis (*n* = 1), rheumatoid arthritis -associated uveitis (*n* = 1)

^***^includes iris ring, hooks and/or or synechiolysis

Better preoperative BCVA was documented in patients with anterior uveitis (*p* = 0.046), recurrent acute uveitis (*p* = 0.045), and patients who underwent standard cataract surgery (*p* = 0.018). Worse preoperative BCVA was noted in patients with a history of chronic uveitis (*p* = 0.045), previous CME (*p* = 0.024). No significant differences in the preoperative BCVA were found in patients receiving systemic treatment (*p* = 0.219) or exhibiting preexisting macular (non-CME) abnormalities (*p* = 0.311). Definitively compromised central visual acuity was established in 9 (12%) patients before the surgery due to due to the structural changes in the retina caused by chronic macular edema.

## Visual acuity according to the anatomical localization and etiology

The postoperative BCVA improved in 86% of patients, remained unchanged in 10%, and deteriorated in 4%. A final BCVA of 0.5 (0.3 logMAR) or better was achieved in 57 (73%) patients. The median change in visual acuity from baseline to final BCVA was − 0.5 logMAR (from − 2.3 to 0.9). A significant correlation was shown between the preoperative and postoperative BCVA (Spearman *r* = 0.521, *p* < 0.01). This correlation remained significant even after the exclusion of patients with permanently decreased central BCVA (*p* < 0.003). The linear regression model has demonstrated that better preoperative BCVA results in better postoperative BCVA (*p* < 0.001). The preoperative and final BCVA did not differ between the genders and age (*p* = 0.355; *p* = 0.370).

**Fig. 1:**
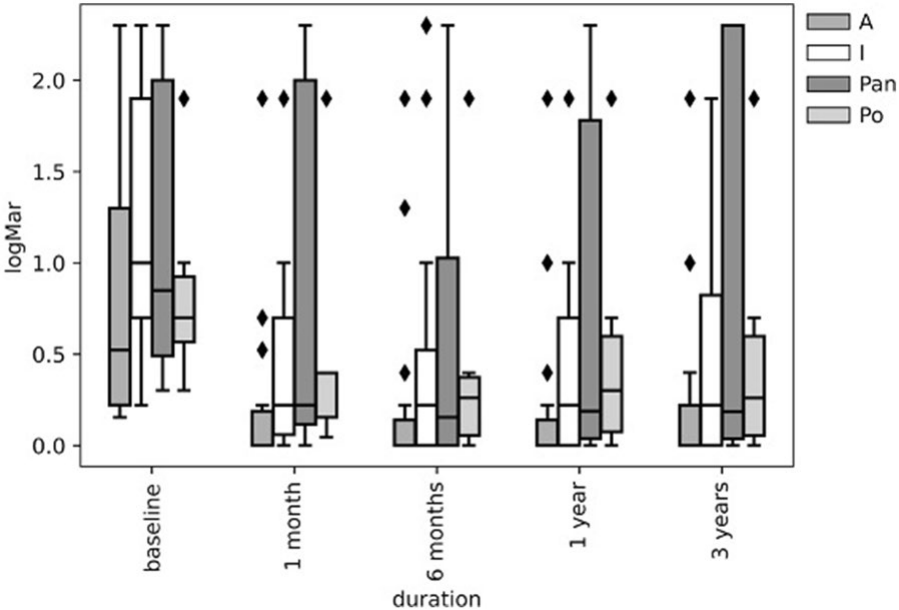
Pre- and postoperative best-corrected visual acuity based on anatomical localization throughout the 3-year follow-up. A, anterior uveitis; I, intermediate uveitis, Pan, panuveitis; Po, posterior uveitis, *filled diamond* represents the outliers

Pre- and postoperative BCVA in relation to anatomical localization of uveitis throughout the 3-year follow-up is shown in Fig. 1 and Table 2, the postoperative BCVA is presented in Table 3. The postoperative BCVA differed between specific anatomical localizations of uveitis (*p* = 0.047), with anterior uveitis having the best visual results (89% of patients with anterior uveitis had BCVA > 0.5). While 62% (5/8) of patients with panuveitis achieved final BCVA > 0.5, the remaining 38% (3/8) had a BCVA of 0.05 or less. Final BCVA between 0.05 and 0.5 was typical for patients with intermediate uveitis, as 22% of patients with intermediate uveitis remained in this range (*p* = 0.008; Table 2).

**Table 2:** Preoperative best-corrected visual acuity according to the anatomical localization of uveitis

Anatomical localization of uveitis	BCVA ≥ 0.5 *N* (%)	0.05 < BCVA < 0.5 *N* (%)	BCVA ≤ 0.05 *N* (%)	Total *N* (%)
Intermediate	4 (11)	17 (46)	16 (43)	37 (100)
Anterior	8 (30)	9 (33)	10 (37)	27 (100)
Panuveitis	1 (13)	4 (50)	3 (37)	8 (100)
Posterior	1 (17)	4 (66)	1 (17)	6 (100)

**Table 3:** Visual outcomes three years after cataract surgery according to the anatomical localization of uveitis

Anatomical localization of uveitis	BCVA ≥ 0.5 *N* (%)	0.05 < BCVA < 0.5 *N* (%)	BCVA ≤ 0.05 *N* (%)	Total *N* (%)
Intermediate	24 (65)	8 (22)	5 (14)	37 (100)
Anterior	24 (89)	2 (7)	1 (4)	27 (100)
Panuveitis	5 (62)	0 (0)	3 (38)	8 (100)
Posterior	4 (67)	1 (17)	1 (17)	6 (100)

*BCVA* best-corrected visual acuity

In our series, no associations between etiological subgroups of uveitis and final BCVA were noted except for FHU, which exhibited better final BCVA (*p* = 0.022). Duration of uveitis had a significant impact on the final BCVA, as chronic uveitis resulted in a worse final BCVA compared to recurrent acute uveitis (*p* = 0.001). The final BCVA in patients who underwent standard cataract surgery and those requiring an additional iris procedure did not differ (*p* = 0.071; *p* = 0.256); however, worse final BCVA was noted in patients with perioperative implantation of dexamethasone (*p* = 0.007). There were no differences in visual outcomes between patients receiving different types of systemic treatments and patients without any systemic therapy (*p* = 0.255). Moreover, a history of previous intraocular surgery or glaucoma had no impact on the final BCVA (*p* = 0.187; *p* = 0.144). Preoperative presence of CME was associated with worse visual outcomes (*p* = 0.006), nevertheless, in our series, the remaining preexisting macular abnormalities (other than CME) had no significant impact on the final BCVA (*p* = 0.070).

## Complications

Development of postoperative CME de novo was seen in 9 (11%) of patients, and an additional 19 (24%) had a history of previous CME before surgery. All the patients with new CME had intermediate uveitis. The patients with intermediate uveitis exhibited the highest rates of postoperative CME (19; 51%; *p* = 0.01). In addition, the presence of postoperative CME was highly associated with preoperative CME (*p* < 0.001) and the intraoperative use of dexamethasone (*p* = 0.009), reflecting the fact that it was used in patients with CME present less than 6 months prior to surgery. In our series, etiology (*p* = 0.30), systemic medication (*p* = 0.082) and iris manipulation during surgery (*p* = 0.218) showed no relationship with the development of postoperative CME. Secondary glaucoma requiring trabeculectomy was noted in 2 (3%) patients within six months after surgery. Other major complications were scarce.

There were 10 (13%) patients with a final BCVA less than 0.01; 9 of them already had definitively limited best-corrected visual acuity before the surgery due to the structural changes in the retina caused by chronic macular edema. In addition, 9 (12%) patients achieved final BCVA between 0.05 and 0.5; notably, 8/9 of them had intermediate uveitis with a preexisting CME, and one had HLA B27-associated anterior uveitis with a CME before the surgery, requiring a dexamethasone implant.

## Discussion

Our study shows that BCVA improved in 86% and the final BCVA of 0.5 or better was achieved in 73% from all patients with uveitis, proving that cataract surgery, albeit technically more challenging, is beneficial for most patients with uveitis. We also demonstrate that chronic uveitis, having CME before surgery and intermediate uveitis were associated with worse visual prognosis. The development of postoperative CME was strongly related to the presence of preoperative CME and was most common in intermediate uveitis.

Our results are in line with previous studies in which the final BCVA of 0.5 or better ranged from 47 to 87% [2–4, 9, 10, 12, 14, 15]. In addition, our results are similar to the previously reported observation of the best visual prognosis in patients with anterior uveitis. [2, 4, 9, 10, 14, 15]. While posterior uveitis and panuveitis are the anatomical entities typically associated with inferior visual results compared to anterior uveitis, [7, 10], it is noteworthy that most patients with all anatomical entities of uveitis achieved BCVA of 0.5 or better. Notably, 65% of patients with intermediate uveitis also achieved a BCVA of 0.5 or better, which is better than previously reported [2, 4, 9, 10, 14, 15] However, it should be noted that a considerable percentage (22%) of patients with intermediate uveitis reached final BCVA in a range less than 0.5 but better than 0.05. Previous studies assessing the outcomes of cataract surgery in intermediate uveitis reported variable outcomes, likely related to higher rates of postoperative CME, which occurred in up to 50% of patients [16]. As the only tertiary referral center in Slovenia, all patients with intermediate uveitis needing surgery in our small country are being referred to our center. Our timing of surgery was carefully chosen, and the treatment was optimized. Hence, we believe the beneficial results might reflect the meticulous pre- and post-operative care. Some authors proposed that a combination of phacoemulsification and pars plana vitrectomy could improve the outcome of cataract surgery in intermediate uveitis [17, 18]. Nevertheless, our results demonstrate that phacoemulsification alone results in satisfactory outcomes for most patients with intermediate uveitis. While vitrectomy in intermediate uveitis might result in a decrease in systemic treatment, our results show that a combination of phacoemulsification and vitrectomy is not needed for a favorable visual outcome. Additionally, the patients are not exposed to possible complications related to pars plana vitrectomy. Novel therapies for the treatment of intermediate uveitis have substantially improved the control of intraocular inflammation and may have also reduced the role of combined lens extraction and vitrectomy in intermediate uveitis. We consider the prevention and aggressive treatment of CME of crucial importance for favorable visual outcomes in our patients with intermediate uveitis.

Delaying the surgery until BCVA significantly drops might result in decreased visibility of the posterior segment, possibly to the point that uveitis-related complications cannot be reliably diagnosed and treated, which may negatively affect the long-term prognosis. This phenomenon might hypothetically explain the strong relationship between preoperative and postoperative BCVA, documented in our series and also shown in the previous reports [5].

Due to the limited number of patients in diverse etiologic groups, reliable assessment according to the causes of uveitis could not be performed. The prevalence of diverse complications, such as increased IOP and development of postsurgical CME, is similar to the previous reports [4, 7, 9, 10, 14, 19]. Zhang et al. [1] have shown that perioperative systemic medications might pose a risk for the development of postoperative CME, however in our series, we could not confirm this observation. The discrepancy could be explained by the differences between the cohorts; Zhang’s cohort included 10 patients with Behcet’s disease and 6 cases of Vogt Koyanagi Harada, which might have affected their outcomes. Earlier studies also reported that iris hook insertion leads to more postoperative inflammation, which also contrasts with our findings, possibly also reflecting differences in the composition of study cohort and the differences in peri- and postoperative treatment [10].

The drawbacks of our study include its retrospective character with all its shortcomings and a limited number of patients. In consequence, the prognosis of specific uveitis etiologies cannot be reliably compared. The strengths were that all our patients were operated by a single surgeon. Our results were obtained with a limited number of patients and should be confirmed in a larger sample of patients with multivariate analysis to identify possible confounders.

## Conclusion

Cataract surgery is a vision-improving procedure for the majority of patients with uveitis. The visual outcome varies between the subtypes of uveitis. Chronic uveitis and intermediate localization, as well as a preexisting CME, were associated with a limited visual prognosis. Therefore, meticulous control of inflammation combined with early and aggressive treatment of CME in patients with intermediate uveitis could result in a more favorable outcome. We do not recommend the postponement of cataract surgery in patients in whom the posterior segment cannot be reliably examined, and in consequence, the possible complications of uveitis cannot be diagnosed and treated.
